# The Prognostic Value and Clinical Use of Myocardial Perfusion Scintigraphy in Asymptomatic Patients after Percutaneous Coronary Intervention

**DOI:** 10.5935/abc.20180199

**Published:** 2018-12

**Authors:** Larissa Franco de Andrade, Ana Carolina Souza, Thais Peclat, Caio Bartholo, Thalita Pavanelo, Ronaldo de Souza Leão Lima

**Affiliations:** 1 Hospital Universitário Clementino Fraga Filho - Universidade Federal do Rio de Janeiro (UFRJ), Rio de Janeiro, RJ - Brazil; 2 Clínica de Diagnóstico por Imagem, Rio de Janeiro, RJ - Brazil

**Keywords:** Myocardial Infarction, Coronary Artery Disease, Myocardial Revascularization, Heart/diagnostic imaging, Percutaneous Coronary Intervention

## Abstract

**Background:**

The role of myocardial perfusion scintigraphy (MPS) in the follow-up of asymptomatic patients after percutaneous coronary intervention (PCI) is not established.

**Objectives:**

To evaluate the prognostic value and clinical use of MPS in asymptomatic patients after PCI.

**Methods:**

Patients who underwent MPS consecutively between 2008 and 2012 after PCI were selected. The MPS were classified as normal and abnormal, the perfusion scores, summed stress score (SSS), and summed difference score (SDS) were calculated and converted into percentage of total perfusion defect and ischemic defect. The follow-up was undertaken through telephone interviews and consultation with the Mortality Information System. Primary endpoints were death, cardiovascular death, and nonfatal acute myocardial infarction (AMI), and secondary endpoint was revascularization. Logistic regression and COX method were used to identify the predictors of events, and the value of p < 0.05 was considered statistically significant.

**Results:**

A total of 647 patients were followed for 5.2 ± 1.6 years. 47% of MPS were normal, 30% were abnormal with ischemia, and 23% were abnormal without ischemia. There were 61 deaths, 27 being cardiovascular, 19 non-fatal AMI, and 139 revascularizations. The annual death rate was higher in those with abnormal perfusion without ischemia compared to the groups with ischemia and normal perfusion (3.3% × 2% × 1.2%, p = 0.021). The annual revascularization rate was 10.3% in the ischemia group, 3.7% in those with normal MPS, and 3% in those with abnormal MPS without ischemia. The independent predictors of mortality and revascularization were, respectively, total perfusion defect greater than 6%, and ischemic defect greater than 3%. Forty-two percent of the patients underwent MPS less than 2 years after PCI, and no significant differences were observed in relation to those who underwent it after that period.

**Conclusion:**

Although this information is not contemplated in guidelines, in this study MPS was able to predict events in asymptomatic after PCI patients, regardless of when they were performed.

## Introduction

The coronary artery disease (CAD) is the leading cause of death in the world.^1^ Percutaneous coronary intervention (PCI) is currently the most commonly used method of coronary artery revascularization in all clinical settings of CAD.^2^ However, despite the technical and pharmacological changes in the last decades, patients undergoing percutaneous revascularization remain at risk of developing cardiovascular events, and the main mechanisms responsible for that are restenosis and progression of atherosclerotic disease.^3,4^

Functional tests, including myocardial perfusion scintigraphy (MPS), are recommended in the evaluation of patients who develop symptoms after PCI.^2,5^ In the presence of significant ischemia, a new revascularization may be proposed. In contrast, in the follow-up of asymptomatic patients, although studies have demonstrated the ability of the MPS to predict future events,^6^ the guidelines do not recommend ordering routine functional tests in a period of less than 2 years, with their performance being acceptable only within this interval in specific subgroups, such as those undergoing incomplete revascularization or with prior silent ischemia, in whom a new approach is feasible.^2,5^

The present study aims to evaluate the association between the clinical and scintigraphic data of asymptomatic patients submitted to MPS after PCI and the occurrence of outcomes; to estimate the prevalence of ischemia and its predictors; to evaluate the indications and MPS timing in these patients; and to compare the characteristics of the patients who underwent MPS befor and after two years of PCI.

## Methods

### Population

**Figure 1:**
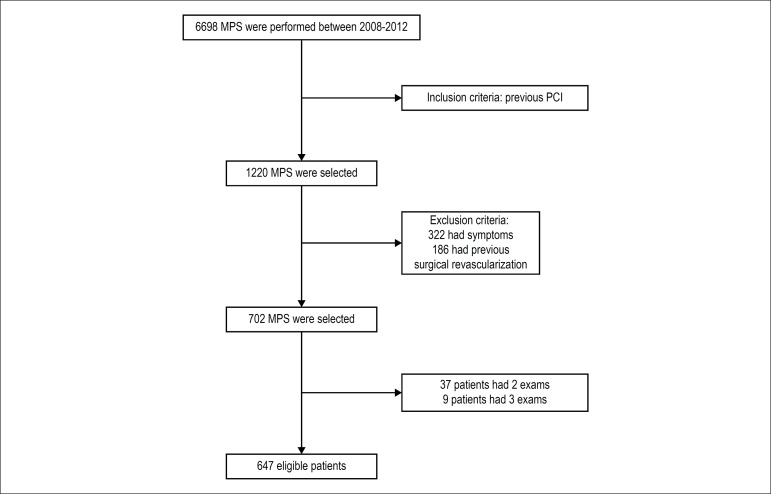
Flow chart of patient selection. PCI: percutaneous coronary intervention; MPS: myocardial perfusion scintigraphy.

Among the 6,698 MPS that were consecutively performed at the Clínica de diagnóstico por imagem in Rio de Janeiro from March 2008 to November 2012, 1,220 patient exams were identified as previously undergoing PCI. Of these, 322 were excluded because the patients had symptoms at the time of the exam, and 186 because they had already undergone a revascularization surgery. Forty-six patients underwent more than one exam in the period and, in those cases, only the first exam was considered. Thus, 647 patients were enrolled in the study, as shown in figure 1.

The study was approved by the Ethics and Research Committee of the Hospital Clementino Fraga Filho, and each patient signed a consent form to include their information in the database, including clinical characteristics and the data of the examination.

### Image protocol

MPS were performed using the 2-day protocol. In the resting phase, a dose of 20mCi ^99^mTc-sestamibi was injected with acquisition of the images after 30 to 40 minutes, and in the stress phase a dose of 20mCi of ^99^mTc-sestamibi was injected during the exercise test or pharmacological stress test, and image acquisition was performed after 15 to 30 minutes. The physical and pharmacological stress protocols were performed as described in a previous study.^7^ MPS images were acquired through the gated-SPECT technique in the Ventri gamma-camera, GE Healthcare.

The exams were classified as normal, or with reversible, fixed or mixed perfusion defects. The semi-quantitative visual analysis was independently performed by two cardiologists with extensive experience, through the standard 17-segment model, in which the quantification of radiotracer uptake was evaluated in each segment, graduated on a scale of 0 to 4, where 0 = normal uptake; 1 = slight reduction of uptake; 2 = moderate reduction of uptake, 3 = severe reduction of uptake; 4 = no uptake.^8^

The values attributed to each of the 17 segments were added in the stress phase, called summed stress score (SSS), and in the resting phase, called summed rest score (SRS). The difference between these two scores is called summed difference score (SDS), and represents the degree of transient reversibility. Abnormal MPS was defined by SSS > 3, and abnormal MPS with ischemia by SDS > 1. SSS and SDS were converted, respectively, into percent of total perfusion defect and ischemic defect by dividing the score by 68 (maximum value of the score) and then multiplying by 100. The ejection fraction (EF) and the left ventricular diastolic and systolic volumes were measured automatically using the software.

### Follow-up

Patients' follow-up was carried out through biannual telephone interviews and application of a standardized questionnaire. Deaths were confirmed consulting the Mortality Information System (SIM) database, and the basic cause of death was identified, and all those included in Chapter IX of the International Classification of Diseases (ICD-10), which comprises the diseases of the circulatory system, were considered cardiovascular. Patients not contacted through telephone calls were considered alive if they were not found in the SIM database, but were considered as loss of follow-up in relation to the other outcomes. Primary endpoints were mortality, cardiovascular mortality, and nonfatal AMI, and surgical or percutaneous revascularization was considered a secondary endpoint.

### Statistical analysis

The analysis was performed in the SPSS statistical package version 23.0. Categorical variables are presented as frequencies and percentages and compared using the chi-square test. Numerical variables are presented as mean and standard deviation, or median and interquartile range, according to the normal distribution pattern assessed by the Kolmogorov-Sminorv test, and compared using Student's t-tests or Mann-Whitney test, as appropriate. Variables with statistical significance in the univariate analysis were included in the multivariate model, using logistic regression and the COX model. Variables with significant correlations among them were excluded from the model. Survival curves of different subgroups were evaluated by the Kaplan Meier estimator and compared by the log-rank test. Statistical significance was defined as a value of p < 0.05.

## Results

A total of 647 patients was included and mean follow-up time was 5.2 ± 1.6 years for mortality analysis. In the analysis of the other outcomes, there was a loss of follow-up of 18 patients and the mean follow-up time was 3.9 ± 1.5 years. The analysis of the demographic characteristics of the population, as shown in Table 1, revealed a mean age of 66.1 ± 10 years and a predominance of males. Arterial hypertension (AH) was the most frequent risk factor, followed by dyslipidemia and diabetes mellitus (DM). Fifty-three percent had a previous history of acute myocardial infarction (AMI). The 18 patients lost at follow-up were compared to 629 contacts and no statistically significant clinical differences were observed between the two groups.

**Table 1:** Characteristics of the study population.

Characteristics	N (%) or mean ± SD
Age (years), mean ± SD	66.1 ± 10
Male gender	464 (72%)
Arterial hypertension	411 (64%)
Dyslipidemia	378 (58%)
Diabetes Mellitus	189 (29%)
Previous AMI	342 (53%)
Current smoking	48 (7%)
Previous smoking	204 (32%)
Family history of CAD	193 (30%)

SD: standard deviation; CAD: coronary artery disease; AMI: acute myocardial infarction.

The median dates for prior PCI were March 2008, and 44% were performed in the context of acute coronary syndrome (ACS). The interval between PCI and MPS was a median of 3 years, and was less than 2 years in 42% of the cases.

Among the MPS indications, a control examination after PCI was the most frequent, reaching 75% of the cases. Incomplete revascularization was the second most common justification (12%), followed by preoperative evaluation (7%). The physical stress protocol was used in 59.5% of the exams. MPS were normal in 47% of patients, abnormal with no ischemia in 23%, and abnormal with ischemia in 30%. Previous AMI and incomplete revascularization as an indication of MPS were independently associated with the presence of ischemia, as shown in Table 2.

**Table 2:** Predictors of ischemia

Characteristics	Univariate analysis OR (95% CI)	p value	Multivariate analysis OR (95% CI)	p value
Age > 70 years	0.36 (0.65 to 1.30)	0,489	0.82 (0.55 to 1.20)	0,309
Male gender	1.13 (0.78 to 1.63)	0,515	1.35 (0.89 to 2.05)	1,155
Diabetes Mellitus	1.22 (0.85 to 1.76)	0,288	1.30 (0.88 to 1.93)	0,179
Previous AMI	2.51 (1.77 to 3.59)	< 0,001	2.87 (1.60 to 5.13)	< 0,001
Previous PCI by ACS	1.90 (1.36 to 2.68)	< 0,001	0.71 (0.41 to 1.24)	0,229
Ejection fraction < 50%	1.52 (1.08 to 2.16)	0,018	1.61 (0.78 to 1.71)	0,454
Pharmacological stress	1.34 (0.95 to 1.89)	0,091	1.22 (0.84 to 1.78)	0,294
MPS indication, incomplete revascularization	3.43 (2,11 to 5.57)	< 0,001	2.99 (1.80 to 4.98)	< 0,001

AMI: acute myocardial infarction; PCI: percutaneous coronary intervention; ACS: acute coronary syndrome; MPS: myocardial perfusion scintigraphy.

During follow-up, 61 deaths were recorded, of which 27 were due to cardiovascular causes. Mortality was higher among patients with abnormal MPS without ischemia, followed by the group with abnormal MPS with ischemia, and less found in the group with normal perfusion. The annual rate of death in each group was 3.3%, 2% and 1.2% respectively. Cardiovascular mortality followed the same pattern of incidence in the groups, with annual rates of 1.4%, 0.9% and 0.5%, respectively.

There were 19 nonfatal AMI and this outcome was also more prevalent among those with abnormal MPS without ischemia compared to the other participants, but without statistical relevance.

A total of 139 revascularizations was documented, 10 patients underwent coronary artery graft bypass surgery, 126 underwent PCI, and 3 underwent both. Among the groups, revascularization was more frequent among patients with ischemia, with an annual rate of 10.3%, and less expressive among patients with normal and abnormal perfusion without ischemia, with an annual rate of 3.7% and 3%, respectively. Data on the occurrence of outcomes according to the perfusion groups are shown in Table 3.

**Table 3:** Outcomes according to perfusion

Endpoints	Normal	Abnormal with ischemia	Abnormal without ischemia	p value
Patients, n	304	193	150	
Death (61)	19 (6,3%)	21 (10,9%)	21 (14%)	0,021
Cardiovascular death (27)	7 (2,3%)	9 (4,7%)	11 (7,3%)	0,064
Patients, n	295	289	245	
Non-fatal AMI (19)	10 (3,4%)	3 (1,5%)	6 (4,1%)	0,855
Revascularization (139)	52 (17,6%)	68 (36%)	19 (13,1%)	< 0,001

AMI: acute myocardial infarction.

**Figure 2:**
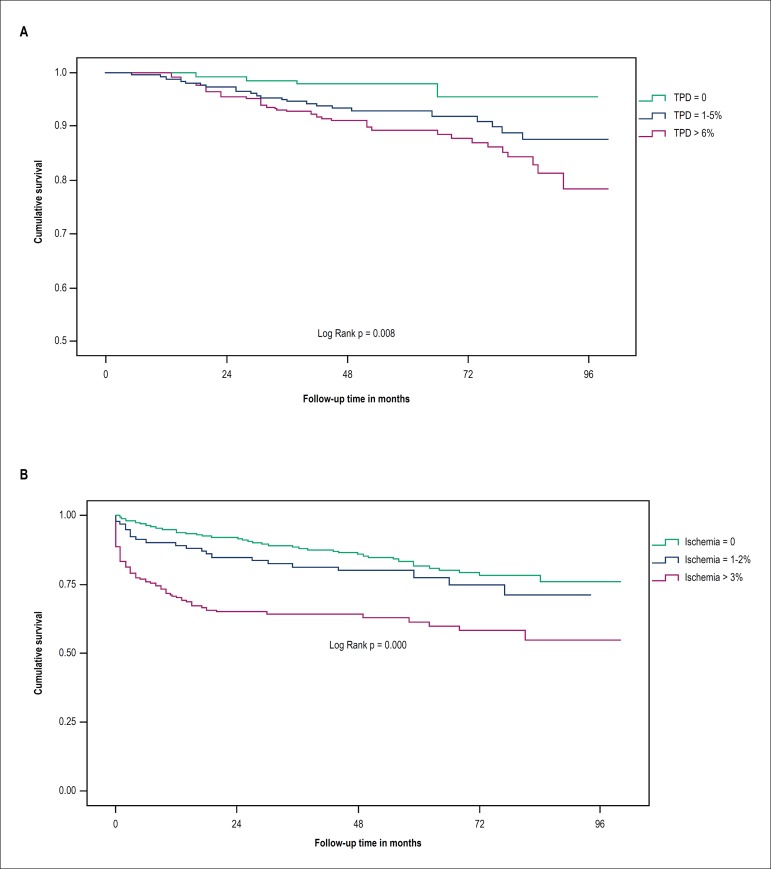
A. A kaplan-Meier survival curve of mortality according to ranges of total perfusion defect (TPD). B. Kaplan-Meier survival curve of revascularization according to ranges of ischemia defect.

In the univariate analysis, including clinical and scintigraphic characteristics, age above 70 years, AH, DM, use of pharmacological stress protocol, indication of preoperative MPS, and total perfusion defect higher than 6% were considered predictors. After multivariate adjustment, with the exception of AH, the other variables emerged as independent predictors of death (Table 4). The Kaplan-Meier survival curve stratified by ranges of total perfusion defect in Figure 2 shows the direct relationship between the extent of the defect and mortality, especially when it reaches values greater than 6%.

**Table 4:** Predictors of mortality

Characteristics	Univariate analysis HR (95% CI)	p value	Multivariate analysis OR (95% CI)	p value
Age > 70 years	4.27 (2.40 to 7.60)	< 0,001	3.40 (1.85 to 6.24)	< 0,001
Arterial Hypertension	2.26 (1.20 to 4.28)	0,010	1.48 (0.73 to 3.00)	0,276
Diabetes Mellitus	3.50 (2,04 to 5.99)	< 0,001	2.37 (1.30 to 4.31)	0,004
Preoperative MPS, indication	3.85 (1.88 to 7.90)	< 0,001	2.25 (1.02 to 4.98)	0,044
Pharmacological stress	4.67 (2.56 to 8.50)	< 0,001	2.51 (1.35 to 4.67)	0,003
TPD > 6%	2.40 (1.40 to 4.08)	0,001	2.33 (1.31 to 4.12)	0,004

MPS: myocardial perfusion scintigraphy; TPD: total perfusion defect.

The independent predictors of revascularization were incomplete revascularization as an indication for MPS, the interval between PCI and MPS of less than 2 years, and the ischemic defect greater than 3%, as shown in Table 5. The Kaplan-Meier curve that was stratified by ischemic defect ranges demonstrates the strong association between the extent of ischemia and the occurrence of the endpoint (Figure 2). The only factor independently associated with cardiovascular mortality was the total perfusion defect greater than 6%, and with non-fatal AMI was the presence of DM.

**Table 5:** Predictors of revascularization

Characteristics	Univariate analysis OR (95%)	p value	Multivariate analysis OR (95% CI)	p value
Age > 70 years	0.78 (0.52 to 1.16)	0,223	0.84 (0.55 to 1.28)	0,419
Diabetes Mellitus	1.30 (0.87 to 1.95)	0,198	1.38 (0.89 to 2.15)	0,145
Previous AMI	1.04 (0.71 to 1.52)	0,823	0.69 (0.45 to 1.06)	0,092
MPS indication, control	0.41 (0.27 to 0.61)	0,000	0.86 (0.46 to 1.63)	0,655
MPS indication, incomplete revascularization	4.80 (2.93 to 7.87)	0,000	3.55 (1.65 to 7.60)	< 0,001
PCI-MPS Time < 2y	1.51 (1.35 to 1.75)	0,001	1.55 (1.36 to 1.83)	0,005
Ischemic defect > 3%	3.07 (2.09 to 4.64)	0,000	2.87 (1.83 to 451)	< 0,001

AMI: acute myocardial infarction; MPS: myocardial perfusion scintigraphy.

When analyzing the group of patients with ischemia at MPS (n = 189), there was a greater presence of males (73% × 63%, p = 0.031), a higher frequency of incomplete revascularization as an indication of the MPS (39% × 14%, p = 0.02) and a higher prevalence of the interval prior PCI-CPM less than 2 years (54% × 30%, p = 0.001) among those submitted to revascularization (36%), compared to the group that did not undergo intervention (64%). The extent of ischemic defect was greater among revascularized patients (7% × 6%, p = 0.162), but different from expected, with no statistical significance. Similarly, mortality was lower among revascularized patients (9% × 12%, p = 0.453), however, with no statistical value.

**Figure 3:**
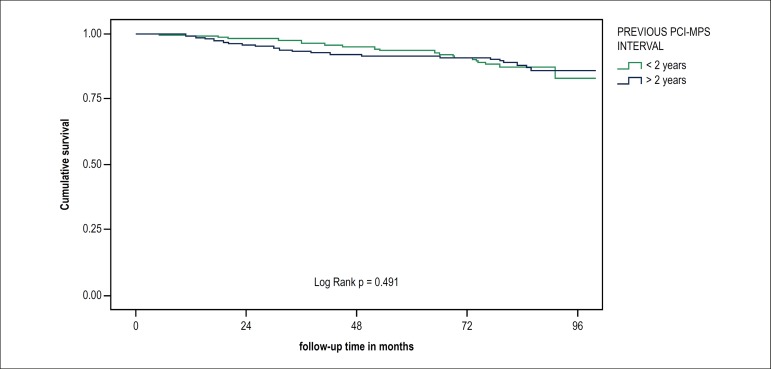
Kaplan-Meier survival curve of mortality according to previous PCI-MPS interval shorter or longer than 2 years.

When comparing the populations that underwent MPS in the interval of less than or more than 2 years after PCI, no significant clinical or scintigraphic differences were observed between them. Mortality in the follow-up period was also similar, as shown in Figure 3.

## Discussion

The use of MPS in the follow-up of asymptomatic patients after PCI has been studied in the last decades. The first studies evaluated the use of MPS in the first 6 months after the procedure;^9-12^ then, some authors tried to establish the use of this functional test later in this subgroup.^13,14^ Most of the publications included patients who underwent MPS after fixed intervals following PCI, ranging from 4 months^12^ to 60 months.^14^ In the current study, this interval varied from days to years, allowing assessment of the prognostic value of MPS when performed at varying intervals after percutaneous revascularization.

In the present study, 647 patients were included and the mean follow-up time was 5.2 years. Previous studies have selected a smaller number of participants, ranging from 196^13^ to 370 patients,^11^ and had less follow-up time, an average of 3 years. Regarding the population characteristics, the predominance of males and the mean age of 66 years were common to other publications, and consistent with data from the literature.^15^ In contrast, the prevalence of comorbidities was quite variable. The current study presented a higher frequency of diabetics. In addition, more than half of the participants had previous AMI, and the prevalence of AH pressure was close to those described above.^11,14^ Such variations can be attributed to the use of different diagnostic definitions of pathologies. On the other hand, they may reflect the selection of populations with different severity profiles, thus with different prognostic aspects.

Despite the lack of information on prior PCI, considering that only 11% of the procedures were performed before 2003, time at which drug-eluting stents were introduced, and that MPS exams were performed in a private clinic in patients with wide access to care, including 30% of diabetics, it is believed that the stents used in previous angioplasties have been almost entirely drug-eluting stents. In previous studies, patients were treated with balloon angioplasty and conventional stent implantation,^9-13^ with the exception of the study by Zellweger et al. in which 69% of the participants were treated with a drug-eluting stent.^14^ Such findings should be taken into account in the interpretation of outcomes, because after the advent of drug-eluting stents, there was a decrease in the incidence of early and late complications of the procedure and, consequently, in the occurrence of events.

Although the current guidelines^2,5,16^ do not indicate routine functional tests, especially in the period of less than 2 years in asymptomatic patients after PCI, in the present study, 42% of MPS were performed within less than 2 years after PCI, and the control examination was the most frequent indication, independent of the period. Similarly, Luca et al.,^17^ in an observational study including 12,380 patients undergoing PCI in Canada from 2004 to 2012, and Shah et al.,^18^ in a study including 21046 patients undergoing percutaneous revascularization between 2004 and 2007 in the USA, observed that 60% and 61%, respectively, underwent at least one functional test within a 2-year period.^17,18^ One possible justification for functional evaluation to remain a frequent clinical practice among asymptomatic patients after PCI is the lack of robust information about the theme that defines the correct management of these patients, and the fact that the current recommendations are based on the opinion of specialists.^2,5,16^

The prevalence of 30% of ischemia among patients was higher than that found in previous studies. Zellweger et al.^14^ detected ischemia in 19% of patients after 60 months of PCI, and Rajagopal et al.^11^ in 23% of those evaluated after 3.9 months. The exception was the study by Galassi et al.,^12^ which included only patients known to undergo incomplete revascularization and, as expected, detected more abnormal perfusions. Similar to previous studies,^9,11^ incomplete revascularization as an indication of MPS and the presence of previous AMI were considered independent predictors of ischemia. In contrast, the presence of DM was not independently associated with ischemia, as described by other authors. One possible explanation, given that all patients are asymptomatic, is the valorization of the presence of comorbidity leading to a higher indication of exams. Seventy percent of the diabetics in this study had indication of control MPS.

Previous studies that analyzed the role of MPS in the follow-up after percutaneous revascularization used the composite endpoint model, which impaired the comparison of the results. It should be noted that the evaluation of events separately, as performed in this study, is important because the endpoints analyzed (death, cardiovascular death, non-fatal AMI and revascularization) have different clinical relevance and occurred at different frequencies in all the studies described.^9-14^

The mortality rate observed was 2% per year, comparable to the rate described by Leon et al.,^19^ in the 5-year follow-up of patients treated with conventional and drug-eluting stents. However, comparing the different perfusion groups, patients with abnormal MPS without ischemia had a mortality rate of 3.3% per year, higher than that found in patients with abnormal perfusion with ischemia and normal perfusion, respectively, 2% and 1.2%. In addition, the extent of the total perfusion defect was independently associated with death when greater than 6%.

In the evaluation of other variables, age greater than 70 years was considered an independent predictor of mortality, which is expected in the natural evolution of coronary disease. Likewise, the presence of DM was associated with a higher risk of death, similar to data in the literature that showed a more diffuse atherosclerotic involvement among diabetics and a higher propensity to develop restenosis after percutaneous intervention, thus leading to greater mortality in the long term.^20^

Acampa et al.^21^ had emphasized that patients undergoing pharmacological stress had a higher age group and a higher prevalence of clinical predictors of ischemia compared to those who underwent physical stress, and, therefore, had a poorer prognosis. Similarly, in the current study, the pharmacological stress protocol was used in 70% of the patients who died and was significantly associated with the endpoint risk. Aspects related to MPS indications also directly influenced the results, with a preoperative examination being associated with a greater chance of death. One possible justification for such finding is the risk inherent to the surgical procedure itself, and the potential severity of the underlying pathology. This variable was not addressed by the other studies already cited.^9-14^

Although they were not included in the multivariate analysis because of a strong correlation with perfusion scores, the presence of prior AMI and lower EF values were more frequently found among those who died, respectively, 69% × 51%, p = 0.009 and 47 ± 16 × 54 ± 12, p = 0.001. Other studies had already demonstrated the impact of ventricular function on survival of patients with coronary artery disease, among which the Coronary Artery Surgery Study (CASS) is highlighted, which observed an inverse relationship between EF and mortality. In this register, survival rates after 12 years of follow-up of coronary arteries disease with EF ≥ 50%, between 35 and 49% and < 35% were, respectively, 73%, 54% and 21% (p = 0,001).^22^

Similar to what was found in the mortality analysis, the outcomes of cardiovascular mortality and non-fatal AMI had a higher incidence in the group with abnormal perfusion without ischemia compared to the others. The absence of statistical significance may be justified by the small number of events, but certainly does not compromise the importance of the findings, especially cardiovascular mortality with p= 0.064, close to what is considered relevant. The only factor independently associated with cardiovascular mortality was the total perfusion defect greater than 6%, and to non-fatal AMI was the presence of DM. Georgoulias et al.,^10^ after an 8-year follow-up of 246 asymptomatic patients undergoing CPM after PCI, also observed that the occurrence of the composite endpoint, cardiovascular death, and non-fatal AMI was greater the greater the extent of the total perfusion defect.

The annual rate of endpoint revascularization was 4.6%, more significant during the 1^st^ year of follow-up compared to that found in subsequent years, 11.9% × 3.4%, respectively. Leon et al.^19^ observed similar results, 20.4% of patients treated with conventional stents, and 11.2% of those treated with drug-eluting stents underwent a new approach in the 1^st^ year of follow-up; then, the annual rate of revascularization was a constant of 3.5% between the 2^(nd)^ and 5^th^ years. In view of these findings, it should be pointed out that, as suggested by Leon et al.,^19^ the events taking place in the first year seem to be related to the initial procedure, with markedly reduced rates of conventional therapy to pharmacological therapy, whereas later revascularizations reflect the progression of disease, with constant rate, regardless of the type of stent used.

Zellweger et al.,^9^ in the follow-up of patients undergoing percutaneous intervention, demonstrated that the cumulative rate of composite outcome was statistically higher among patients with ischemia than those without ischemia at MPS, and revascularization corresponded to 65% of these events. Similarly, Galassi et al.,^12^ in a cohort consisting of asymptomatic patients submitted to incomplete percutaneous revascularization, reported that 42% of the participants performed a new approach at the mean follow-up of 33 months, and that the extent of ischemia in the MPS performed 4 to 6 months after the procedure was a predictor of this outcome.

In the current study, in addition to the presence and extent of ischemia, incomplete revascularization as an indication of MPS and the interval between percutaneous intervention and MPS before 2 years were also significantly associated with revascularization. These results suggest that the decision for the new approach was probably influenced by the initial procedure. This hypothesis was reinforced when it was observed that in the group of patients with ischemia, among the 36% who underwent the new revascularization, there was a predominance of males and, again, incomplete revascularization as an indication of MPS, and of the interval between PCI and MPS of less than 2 years.

The extent of ischemia was also higher among those referred to repeated revascularization, but, unlike expected, this finding was not statistically significant. It is possible that in some cases the presence and not the extent of ischemia has been a variable with greater impact in the decision making for revascularization. Regarding male gender, it shows a higher prevalence of coronary disease and greater precocity in the event occurrence; this may have contributed to the valorization of the findings and the indication of approach in the patients of this gender.

Aldweib et al.,^23^ in the evaluation of 769 asymptomatic patients previously undergoing PCI with ischemia in MPS, subsequently referred for drug therapy or revascularization, found greater extent of ischemia and greater presence of DM among the revascularized patients. Different from the current study, the interval between PCI and MPS was similar between the groups and the presence of incomplete revascularization was not mentioned. After an average of 5.7 years, mortality rates were similar in the two treatment groups (p = 0.84).

In our study, the mortality among those who revascularized was lower than those who received clinical treatment (9% × 12%), but with no statistical significance. Although this study was not designed for this purpose, and the possible impairment of the statistical analysis due to the small number of events, it is worth questioning whether the patients referred to the new revascularization would not be at greater risk and after the procedure had this risk matched to the ones targeted for clinical treatment.

Although the current literature recommends the functional evaluation of asymptomatic patients after PCI only after 2 years,^2,5,16^ in the present study, the clinical features and results of MPS, including perfusion findings, were similar among patients who underwent MPS in the smallest interval and in the one greater than 2 years. In this selected population, the delimitation currently recommended in 2 years did not separate distinct populations.

Although incomplete revascularization is a satisfactory solution when the culprit lesion is identified and has a favorable anatomy for percutaneous approach, especially in the context of ACS, patients with remaining lesions need to be monitored and stratified, regardless of the presence of symptoms. In the present study, in this scenario MPS was shown to be a tool used in clinical practice capable of providing incremental prognostic information about the occurrence of events, directly interfering with the decision to indicate new revascularization.

Previous studies^13,21^ that performed MPS in the follow-up of patients previously undergoing percutaneous revascularization described an excellent prognosis associated with normal perfusion, with an annual event rate of less than 1%. Similarly, in the current study, at the mean follow-up of 5 years, among patients with normal perfusion, the annual mortality rate was 1%, and cardiovascular mortality was 0.5%, characterizing this group as low risk.

### Limitations

This is a single center retrospective study in which the patients were referred to the clinic for MPS at the recommendation of their attending physician. Therefore, extrapolation of the results should be done with caution.

Another limitation is the lack of information on the type of stent used in the prior revascularization procedure in most patients. However, considering that only 11% of the procedures were performed before 2003, at which time drug-eluting stents were introduced, and that the population was selected in a private clinic that mainly serves complementary health users with a DM prevalence of 30 %, it is believed that the stents used have been mostly drug-eluting ones.

## Conclusion

In this study, MPS performed in asymptomatic patients after various periods of PCI was able to provide future prognostic information, the extent of the total perfusion defect was associated with a higher mortality rate and cardiovascular death, the presence and extent of ischemia were associated with higher rate of revascularization, while normal perfusion lead to an excellent prognosis with a low rate of events at the mean follow-up of 5 years.

In spite of the recommendations of the guidelines, in this study, 42% of MPS were performed within less than 2 years after PCI and no relevant clinical differences were observed in relation to those who performed after this period.
